# Sporadic Creutzfeldt-Jakob Disease in a Patient With Multiple Sclerosis: A Case Report

**DOI:** 10.7759/cureus.26879

**Published:** 2022-07-15

**Authors:** Luna Paudel, Suren Limbu, Lu Yu, John A Voss, Marvin Koss, Michael Vertino, Scott F Ulberg

**Affiliations:** 1 Psychiatry, Upstate University Hospital, Syracuse, USA; 2 Psychiatry, B.P. (Bishweshwar Prasad) Koirala Institute of Health Sciences, Dharan, NPL; 3 Neurology, Upstate University Hospital, Syracuse, USA

**Keywords:** behavioral changes, mri images, relapsing-remitting multiple sclerosis, prion diseases, sporadic creutzfeldt–jakob disease

## Abstract

Sporadic Creutzfeldt-Jakob disease (CJD) is a rare neurodegenerative condition and a human prion disease. Rapid progressive dementia, myoclonus, visual disturbances, cerebellar signs, and pyramidal/extrapyramidal symptoms are observed in such patients. However, these are non-specific symptoms and can manifest in a variety of other conditions. The occurrence of sporadic CJD in a patient with multiple sclerosis (MS) is rare. This is the case of a 54-year-old man on natazulimab for MS who developed rapid neurocognitive changes along with visual changes, imbalance issues, and mood changes. Diagnosis of sporadic CJD (sCJD) was confirmed through clinical features, physical examination and electroencephalogram findings, cerebral spinal fluid analysis, and later magnetic resonance imaging findings. sCJD with MS being a rare phenomenon, its recognition requires a high index of suspicion, careful chronological evaluation of the patient’s symptoms, and relevant investigations that can aid in reaching the diagnosis.

## Introduction

Creutzfeldt-Jakob disease (CJD) is one of the rare neurodegenerative disorders caused by an abnormal isoform of a cellular glycoprotein known as the prion protein. Types of CJD include sporadic CJD (sCJD), familial, variant, and iatrogenic CJD [1]. The progression of this disease is rapid and usually results in death within a year of its onset. As the definitive diagnosis of sCJD can be done only by postmortem brain biopsy, diagnosis is most challenging due to its rarity, low index of clinical suspicion, and nonspecific clinical features [2]. Furthermore, the presence of this rare disorder in other neurological disorders like multiple sclerosis (MS) can pose greater diagnostic difficulties. We report a unique case of an elderly man who was under treatment for MS for several years and later suffered from sCJD.

## Case presentation

A 54-year-old Caucasian man with a college education who worked as a school business administrator came to visit our emergency department. He was under treatment with natalizumab for the diagnosis of relapsing-remitting (R/R) MS since March 2013. In early 2022, he started experiencing some cognitive decline. His wife reported him as having difficulties with simple mathematics calculations, recalling recent events, and change in mood with increasing agitation. He also complained of vision problems with decreased color perception and alterations in hearing.

During the same time, he reported falls due to gait imbalance. He found himself unable to complete a computer assignment that he had been doing for the past 20 years. He reported crying to sleep one night. All these incidents took a significant toll on his mental health making him more irritated, frustrated, and very short with others in his interactions. These were all out of his character.

He remembered having brain fog and recalled driving, unknowingly tailgating the automobile in front of him. His wife also noticed that he could not use his phone and kept forgetting his age and time. The worsening of all these symptoms compelled him to seek medical advice. He was initially treated with high-dose oral steroid medication as relapse of MS was made as the working diagnosis. The treatment failed to relieve these symptoms and worsened his mood swings.

Upon evaluation at our institution, he was awake, alert, and responsive to the interviewer’s questions. Attention was assessed with a digit span test, which was normal. The serial subtraction test for concentration was also intact. The patient was oriented to part of the day, date, month, and self in the emergency department, but not to location and year. He was agitated, hyperventilating, and anxious about the next treatments that could help him. After admission, he had considerable objective cognitive decline assessed through a cognitive scale. At times, he could not perform the simple task of serial 7 subtractions. His orientations to self and others fluctuated significantly. Mood changes were frequently observed from depressed affect to euphoria. His insight and judgment were preserved and, hence, was worried about missing his children and their upbringing.

During the work-up, all the infectious diseases including cerebrospinal fluid (CSF) infections and autoimmune diseases were ruled out with extensive investigations. Video electroencephalography (vEEG) showed lateral periodic discharges on the right parietal-occipital region.

**Figure 1:**
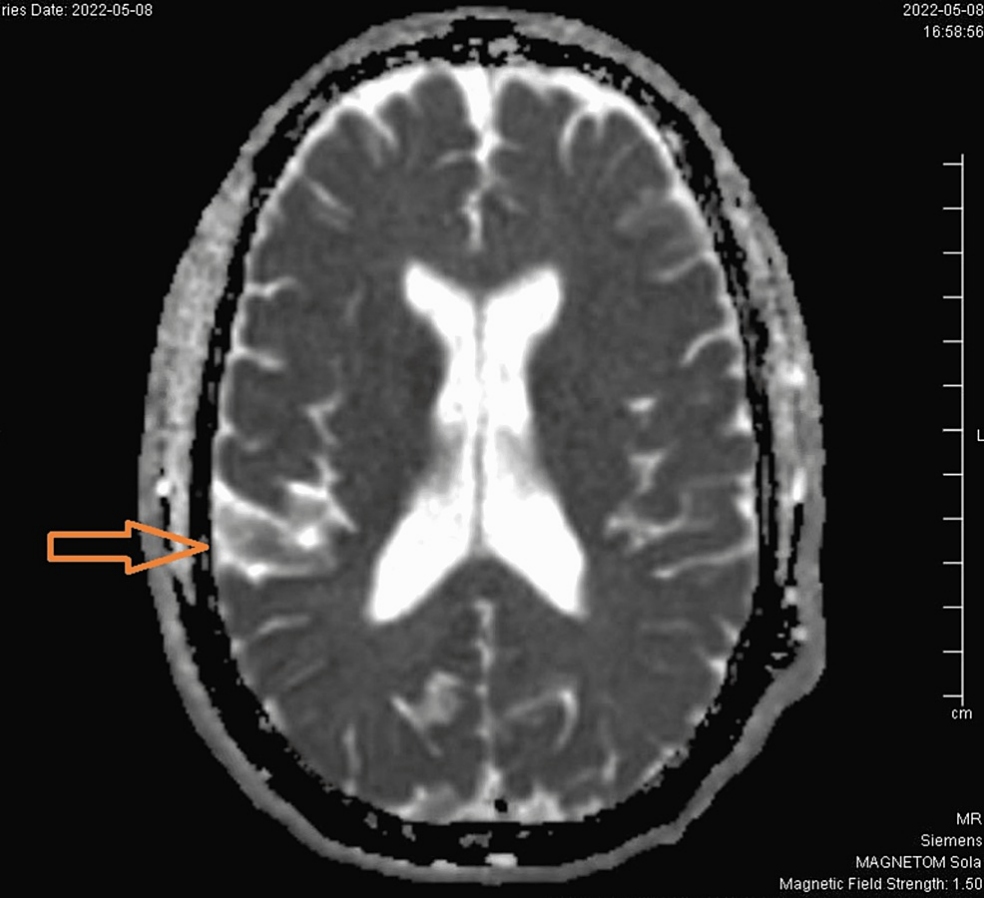
Temporoparietal ribboning of the gyri seen in the right side DWI image DWI: diffusion-weighted imaging

**Figure 2:**
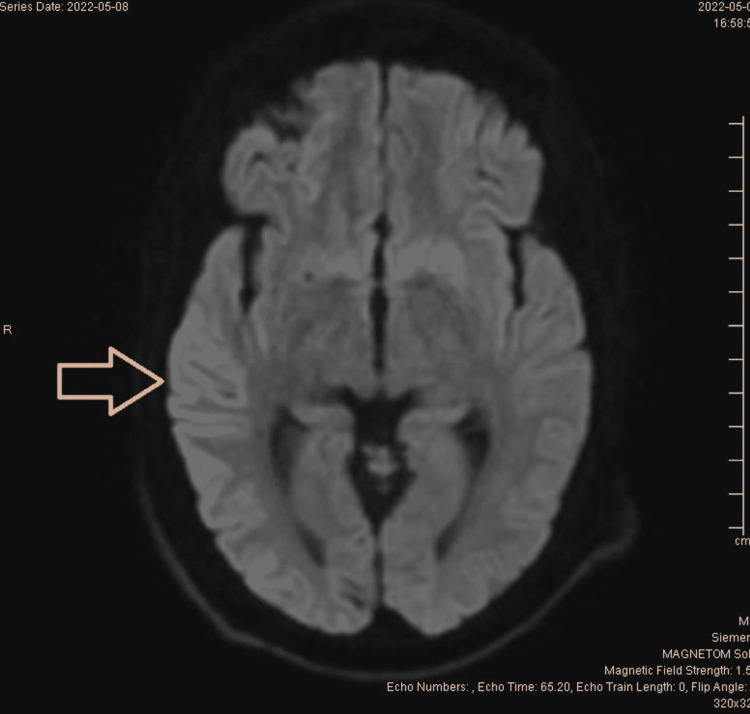
Hyperintensity seen in the right temporoparietal region

Magnetic resonance imaging (MRI) of the brain initially appeared normal but upon further review and discussion with a neuroradiologist, diffusion-weighted imaging (DWI) showed temporoparietal ribboning (Figure 2) and increased signal intensity in bilateral caudate (Figure 1). Unchanged patchy area of T2 and fluid-attenuated inversion recovery (FLAIR) prolongation was observed in the left parietal lobe cortical lesion and bilateral centrum semiovale extending into the cerebral peduncle bilaterally. No space-occupying lesions were noted. There was no abnormal enhancement or restricted diffusion to suggest active demyelinating lesions. Cervical MRI and thoracic spine were negative for any pathology. To investigate further, brain MRI with contrast and without contrast was re-ordered, which showed more prominent DWI changes in the right temporoparietal region and bilateral caudate. Considering the patient's time course, rapidly progressive neurological decline, and MRI findings, CSF analysis for RT-QuIC and 14-3-3 protein was performed.

RT-QuIC (CSF) and 14-3-3 protein (CSF) came out to be positive. T-tau protein (CSF) was elevated (2492 pg/ml) suggestive of neuronal degeneration. The patient’s clinical features along with MRI DWI findings and positive CSF analysis all pointed to "probable sCJD" as per CDC’s Diagnostic Criteria for Creutzfeldt-Jakob Disease (CJD), 2018. The patient’s condition deteriorated rapidly. While being planned for hospice care, the patient passed away, almost two months after his onset of symptoms.

## Discussion

Our patient presented with rapid neurocognitive changes along with visual changes, imbalance issues, and mood changes. Clinical features in sCJD can be non-specific and such presentation may have multiple differential diagnoses. Extensive work-up to rule out other differential diagnoses like infectious, metabolic, and other neurodegenerative conditions were done in our patient. For diagnosis of sCJD, brain biopsy is considered the gold standard. However, antemortem probable sCJD can be diagnosed based on criteria provided by WHO, which include progressive dementia and at least two of the following: myoclonus, visual or cerebellar disturbances, pyramidal/extrapyramidal symptoms, and akinetic mutism. Work-up such as the presence of typical EEG, a positive 14-3-3 CSF assay, and a clinical duration to death <2 years are other criteria [3]. Our patient had rapidly progressive dementia, which is commonly observed in the sporadic form of CJD unlike in variant CJD or inherited prion disease like Gerstmann-Sträussler-Scheinker (GSS) syndrome, or fatal familial insomnia (FFI), where there may be other predominant clinical features [4]. Familial CJD (fCJD) has similar clinical features as sCJD; however, it has an earlier age of onset and a slower disease course (a few months to four years) [5]. Our patient had visual and cerebellar disturbances but did not have myoclonus and extrapyramidal symptoms. Atypical presentation, like in our patient, observed in sCJD can result in a diagnostic dilemma [6]. Akinetic mutism can only be seen in later stages of illness.

CDC criteria for sCJD are much similar to that of WHO but also include MRI findings of high signal abnormalities in the caudate nucleus and/or putamen, especially in DWI and FLAIR images [7]. Currently, MRI plays a valuable role in the initial diagnosis of CJD where complete clinical features may only be appreciated in the late part of the illness. The cortical ribbon sign in MRI DWI, which is a ribbon-like signal hyperintensity of cerebral cortical gyri, is the most striking feature consistent with sCJD [8]. MRI DWI of our patient also showed prominent signal intensity in the right temporoparietal region suggestive of cortical ribbon sign along with hyperintensity of bilateral caudate. However, MRI changes in sCJD can be easily missed in the first instance as in our case mainly because of subtle changes, unfamiliarity with changes by clinicians, the rarity of illness, and degrading of MRI image quality due to movement artifacts, etc [9]. Positive 14-3-3 CSF assay and EEG finding of periodic waves further support the diagnosis of sCJD in the patient.

MS, which is itself a chronic, progressive, demyelinating disease of the CNS, can present with varieties of neurocognitive problems. It is estimated that around 40-60% of patients with MS have cognitive problems at some time in their disease course [10]. Since our patient was on natalizumab for treatment of MS, a relapse of MS was high on the differential. However, the primary debilitating cognitive dysfunction in MS tends to be rare, without accompanying disabilities in motor, sensory, or cerebellar function [11]. MS with cortical demyelination with severe progressive dementia has been increasingly recognized; however, in our case, MRI showed no increased intensity in the brain or spinal cord.

Reaching the diagnosis of sCJD in a patient with MS was tedious but equally challenging as it required a high index of suspicion with consideration of various other differential diagnoses. There is a paucity of reports of sCJD occurring in a patient with MS; however, one case similar to ours was reported previously in which a patient with MS, who was on immunomodulatory treatment, was later diagnosed with sCJD [12]. Furthermore, some authors claim that sCJD shows various clinical, genetic, pathological, and immunological features through which it resembles a severe form of MS [13].^ ^However, robust evidence or findings to support this has been lacking.

## Conclusions

Co-occurrence of sCJD with MS, being a rare phenomenon, can pose a diagnostic dilemma. A high index of suspicion, careful chronological evaluation of the patient’s symptoms, EEG findings, and CSF analysis along with the appreciation of MRI DWI changes can all aid in reaching the diagnosis.

## References

[1] Groveman BR, Foliaki ST, Orru CD, Zanusso G, Carroll JA, Race B, Haigh CL (2019). Sporadic Creutzfeldt-Jakob disease prion infection of human cerebral organoids. Acta Neuropathol Commun.

[2] Kwon GT, Kwon MS (2019). Diagnostic challenge of rapidly progressing sporadic Creutzfeldt-Jakob disease. BMJ Case Rep.

[3] (1998). Global Surveillance, Diagnosis And Therapy Of Human Transmissible Spongiform Encephalopathies: Report Of A WHO Consultation, Geneva, Switzerland, 9-11 February 1998. https://apps.who.int/iris/bitstream/handle/10665/65516/WHO_EMC_ZDI_98.9.pdf?sequence=1&isAllowed=y.

[4] Manix M, Kalakoti P, Henry M, Thakur J, Menger R, Guthikonda B, Nanda A (2015). Creutzfeldt-Jakob disease: updated diagnostic criteria, treatment algorithm, and the utility of brain biopsy. Neurosurg Focus.

[5] Gambetti P, Kong Q, Zou W, Parchi P, Chen SG (2003). Sporadic and familial CJD: classification and characterisation. Br Med Bull.

[6] Head MW (2013). Human prion diseases: molecular, cellular and population biology. Neuropathology.

[7] (2022). CDC’s Diagnostic Criteria for Creutzfeldt-Jakob Disease (CJD), 2018. Classic.

[8] Abdulmassih R, Min Z (2016). An ominous radiographic feature: cortical ribbon sign. Intern Emerg Med.

[9] Carswell C, Thompson A, Lukic A (2012). MRI findings are often missed in the diagnosis of Creutzfeldt-Jakob disease. BMC Neurol.

[10] Rao SM (1995). Neuropsychology of multiple sclerosis. Curr Opin Neurol.

[11] Staff NP, Lucchinetti CF, Keegan BM (2009). Multiple sclerosis with predominant, severe cognitive impairment. Arch Neurol.

[12] Gabelić T, Habek M, Zerr I, Gawinecka J, Pavlisa G, Brinar VV (2011). Sporadic CJD in a patient with relaplsing-remitting multiple sclerosis on an immunomodulatory treatment. Acta Neurol Belg.

[13] Ebringer A, Rashid T, Wilson C, Boden R, Thompson E (2005). A possible link between multiple sclerosis and Creutzfeldt-Jakob disease based on clinical, genetic, pathological and immunological evidence involving Acinetobacter bacteria. Med Hypotheses.

